# A novel adaptive filter with a heart-rate-based reference signal for esophageal pressure signal denoising

**DOI:** 10.1007/s10877-023-01116-z

**Published:** 2024-02-04

**Authors:** Yu Qin, Zhiwen Huang, Xiaoyong Zhou, Shuiqing Gui, Lihong Xiong, Ling Liu, Jinglei Liu

**Affiliations:** 1 Shenzhen Institutes of Advanced Technology, Chinese Academy of Sciences, Shenzhen, 518055 China; 2 Shenzhen Mindray Bio-Medical Electronics Co. Ltd., Shenzhen, 518057 China; 3 Department of Critical Care Medicine, Shenzhen Second People’s Hospital, The First Affiliated Hospital of Shenzhen University, Shenzhen, 518025 China; 4 Jiangsu Provincial Key Laboratory of Critical Care Medicine, Department of Critical Care Medicine, Zhongda Hospital, School of Medicine, Southeast University, Nanjing, China

**Keywords:** Adaptive filter, Esophageal pressure, Cardiogenic oscillations, Denoising, Heart rate, Lung compliance

## Abstract

Esophageal pressure (Peso) is one of the most common and minimally invasive methods used to assess the respiratory and lung mechanics in patients receiving mechanical ventilation. However, the Peso measurement is contaminated by cardiogenic oscillations (CGOs), which cannot be easily eliminated in real-time. The field of study dealing with the elimination of CGO from Peso signals is still in the early stages of its development. In this study, we present an adaptive filtering-based method by constructing a reference signal based on the heart rate and sine function to remove CGOs in real-time. The proposed technique is tested using clinical data acquired from 20 patients admitted to the intensive care unit. Lung compliance ( QUOTE ) and esophageal pressure swings (△Pes) are used to evaluate the performance and efficiency of the proposed technique. The CGO can be efficiently suppressed when the constructional reference signal contains the fundamental, and second and third harmonic frequencies of the heart rate signal. The analysis of the data of 8 patients with controlled mechanical ventilation reveals that the standard deviation/mean of the QUOTE is reduced by 28.4–79.2% without changing the QUOTE and the △Pes measurement is more accurate, with the use of our proposed technique. The proposed technique can effectively eliminate the CGOs from the measured Peso signals in real-time without requiring additional equipment to collect the reference signal.

## Introduction

Pleural pressure (Ppl) is the pressure within the pleural cavity. It is an important index for analyzing the respiratory mechanism. One of the most common and minimally invasive techniques for indirect estimation of Ppl is based on esophageal pressure (Peso) measurements [1–3]. Thus, the measurement of Peso can be used to estimate variables of clinical importance, such as lung compliance ( QUOTE ), work of breathing, transmural vascular pressure [2], intrinsic positive end-expiratory pressure, respiratory effort, and chest-wall compliance [4]. Furthermore, it facilitates the detection of patient-ventilator asynchrony [5–7], thereby supporting specific diagnoses and interventions [2].

The method of Peso measurement uses catheters with air-filled or liquid-filled balloons (primarily in neonates) [8], or small transducers placed in the esophagus [9]. The most common measurement technique involves the insertion of an esophageal balloon, which is coupled to a long and thin catheter inflated with an ideal volume of air into the lower third of the intrathoracic esophagus [2, 10, 11].

Respiratory activity is the primary cause of change in the esophageal pressure. However, as the esophageal balloon placement is close to the vicinity of the heart, esophageal pressure measurements are sensitive to cardiogenic oscillations (CGOs). Thus, the accuracy of the Peso measurements is affected by cardiac artifacts [12–15]. To obtain accurate Peso measurements, the CGOs must be eliminated from the detected Peso signals. However, this is very challenging because the bandwidths of the Peso and CGO signals are very close.

The Peso signal's bandwidth ranges from 0.17 to 0.67 Hz [1], whereas the bandwidth of the CGOs ranges from 0.8 to 4 Hz [16]. The upper band limit of the Peso signal and the lower band limit of the CGO signal differ by only 0.13 Hz. Cheng et al. reported that the harmonics of the Peso and CGO signals, particularly the second harmonic, were one of the main components [17]. Thus, the Peso signal may overlap with the CGO signal in the frequency domain. Direct use of standard filters on CGO-contaminated Peso signals, such as bandpass and band-stop filters with set cut-off frequencies, may not yield adequate denoising performance [1, 17].

Schuessler et al. suggested an approach based on adaptive filtering for eliminating CGOs from the Peso signal [18]. The method described in [18] used the electrocardiogram (ECG) and Peso signals from the same individual and employed a linear dynamic filter to produce an artifact-free Peso signal. However, their method required a two-sided 256-order finite impulse response filter, which caused a 1.28 s delay at a sampling rate of 100 Hz. Additionally, the adaptive filters require (1) 60 s to adapt to the heart rate, and (2) consecutive 10 stable and clean respiration efforts. These findings indicate that their method is unsuitable for short-duration Peso signals, and real-time noise reduction is not possible.

Cheng et al. proposed a modified adaptive noise cancellation (MANC) technique for denoising the Peso signals by utilizing an airflow signal as a reference to estimate the CGOs [1, 17]. They demonstrated the effectiveness of the MANC technique in separating the CGOs from the Peso signals based on Brown–Norway rat experiments. However, since the bandwidths of both the Pes and CGO signals in humans are lower than those in rats, and the situation in the clinic is more complicated as airflow and Pes signals are not always coupled, further research is needed to assess the suitability of this method for intensive care unit (ICU) patients.

Graßhoff et al. proposed a template subtraction method for the reduction of the amplitudes of the CGOs on Peso signals based on the modification of the adaptive filtering approach presented by Schuessler et al. [4]. Additionally, this approach needs an electromyographic (EMG) signal as a reference signal to estimate the CGOs.

Mukhopadhyay et al. recently proposed a singular spectrum, analysis-based, and data-driven technique to eliminate CGOs from Peso signals; it takes advantage of the intrinsic periodicity and morphological characteristics of the Peso signal [19]. This method does not require a reference signal. Their results revealed that the proposed denoising technique can efficiently remove the CGO noise with adequate robustness by testing 75 clinical esophageal pressure signals and 1800 synthesized signals from pure esophageal pressure and real CGOs. It is a singular value decomposition based signal denoising technology and can be easily adapted for denoising other biomedical signals such as electrocardiograms and photoplethysmograms [20], which exhibit periodic or quasi-periodic nature.

Blind Signal Separation (BSS) is an effective denoising method that has found widespread application in the field of biomedical signal processing. It is used to separate and eliminate unwanted noise or interference from biomedical signals, including ECG [21], electroencephalograms (EEG) [22], and EMG [23], without prior knowledge of the noise sources. However, this approach requires multiple sensors for the application and, independence between the desired and undesired components [24]. It's worth mentioning that Peso signals are typically acquired using a single sensor, making this method less suitable for denoising Peso signals.

All the techniques mentioned above require an additional reference signal for estimating the CGOs (with the exception of the Mukhopadhyay method), such as ECG, EMG, or airflow. The acquisition of extra ECG and EMG signals requires additional equipment, making the clinical system more complicated, increasing the patients’ cost, and possibly impeding the patients’ comfort. In the Mukhopadhyay method, the minimum length of the esophageal pressure signal needed to include all the respiratory efforts, i.e., the inspiratory and expiratory processes, might limit real-time denoising applications.

To overcome the disadvantages of the aforementioned methods, herein, we develop a real-time technique to eliminate CGOs from the measured Peso signals based on adaptive filtering and heart rate, without requiring additional equipment for reference signal acquisition. The performance of the proposed technique is tested by using frequency and remnant noise analyses on clinical data collected from 20 patients in a respiratory ICU. Finally, we demonstrate the effects of the proposed technique based on the estimates of and esophageal pressure swings (△Pes) in these patients.

## Materials and methods

### Adaptive filtering

**Fig. 1:**
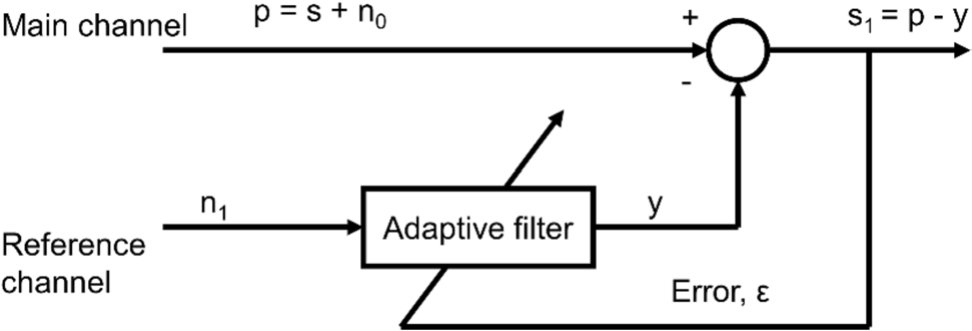
Schematic of the conventional adaptive noise cancellation system

The schematic of the conventional adaptive noise cancellation system [25, 26] is shown in Fig. 1. It uses a noisy signal as the processing object and suppresses or attenuates the noise to improve the signal-to-noise ratio quality of the output signal. As shown in Fig. 1, the adaptive noise cancellation system has two channels, namely the main and the reference channels. The main channel’s input is the noisy signals (p) detected from the transducer which comprise the clean (s) and the noise signals (n0), i.e., the CGO-contaminated Peso signals in this study. The reference channel needs a reference signal (n1) related to the noise n0 as its input. The reference signal goes through an adaptive filtering process, and the signal (y) is the output, which is close to the noise n0. To achieve this, the adaptive filter is adjusted by an adaptive filtering algorithm, such as the least-mean-squares (LMS) algorithm based on the error ε. Thus, we can obtain a denoised signal s1 by using the noisy signal p, which is subtracted from signal y by a subtractor.

### Proposed adaptive filter

**Fig. 2:**
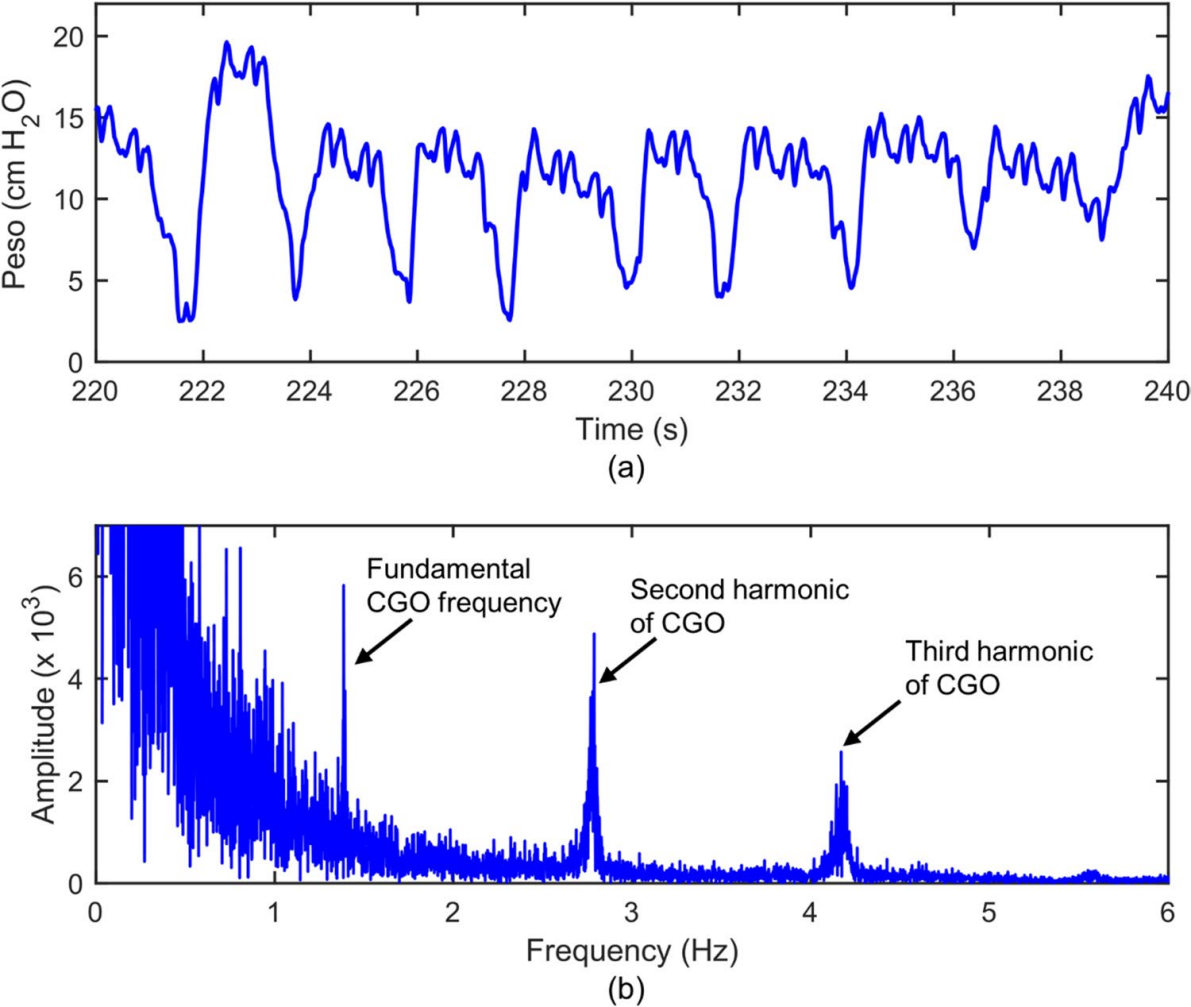
**a** CGO-contaminated Peso signal (original signal) and **b** its frequency spectrum

It is well known that CGOs are correlated to the heart rate [1, 4, 17–19]. This can be demonstrated from the frequency domain of the CGO-contaminated Peso signals calculated based on the Discrete Fourier transform, as shown in Fig. 2b. In this sample signal, the heart rate of the patient was 84 beats per minute (1.4 Hz). In the frequency domain, the maximum peak value, which appeared at 1.4 Hz, was considered as the fundamental frequency of the CGO signal. The second and third harmonic frequencies of CGO were 2.8 and 4.2 Hz, respectively. If we can suppress the frequency, such as the fundamental, and the second and third harmonics related to the CGO signals in the frequency domain, the CGOs would be eliminated from the measure Peso signals. Therefore, we proposed the construction of a CGO-related reference signal based on the heart rate by using the sine function. The heart rate was defined as the average adjacent temporary heart rate over 10 s, where the temporary heart rate was obtained by measuring the reciprocal of the distance between the peak of two adjacent CGO-related noises from the Peso signal. Thus, the heart rate could be obtained directly from the Peso signal, i.e., no additional equipment was required in this study.

**Fig. 3:**
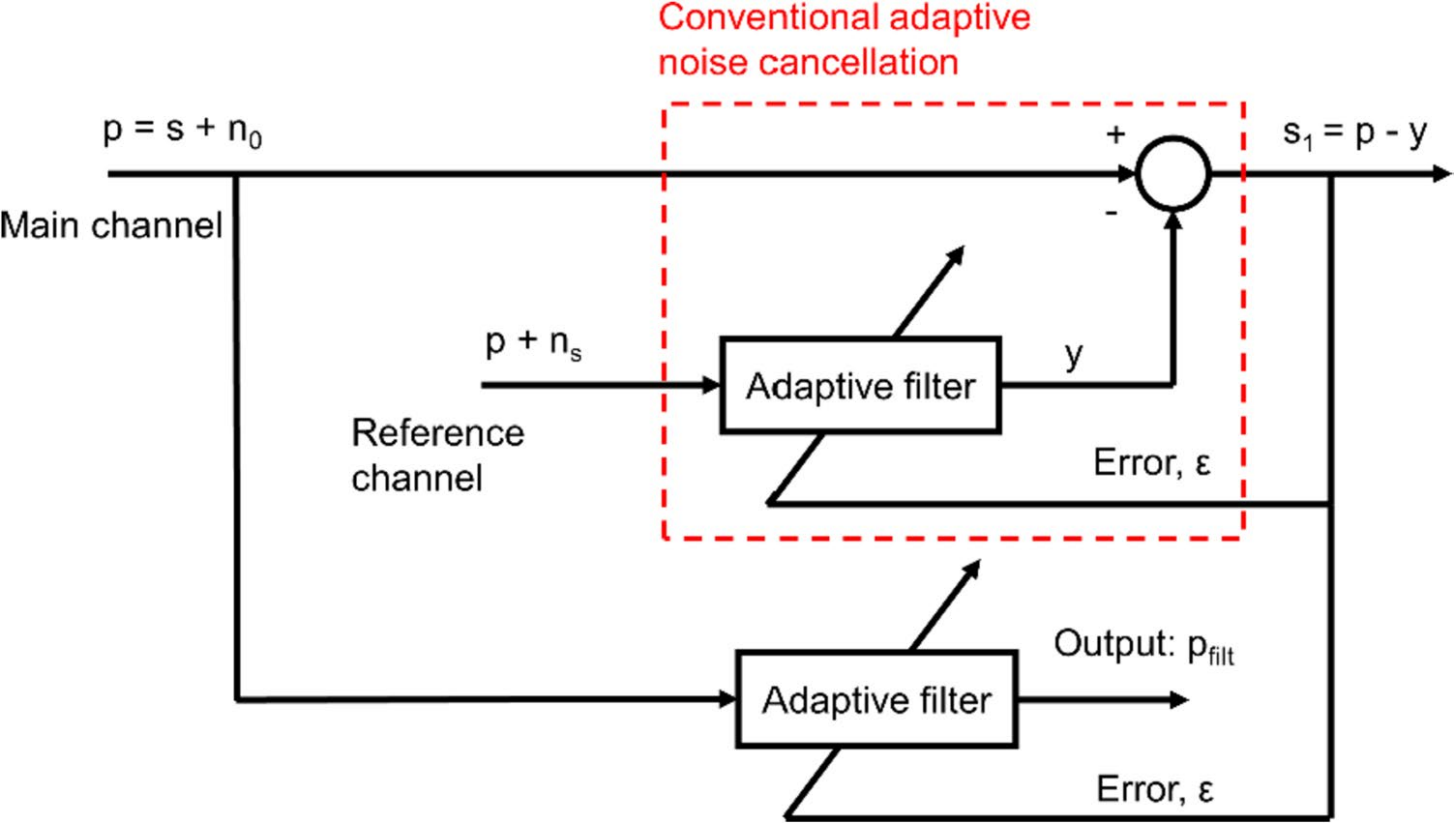
Schematic of proposed adaptive noise cancellation system (*p* measured Peso signal, *s* clean Peso signal, *n*_*0*_ cardiogenic oscillation (CGO) signal, *n*_*s*_ constructional signal, *P*_*filt*_ Denoised Peso signal)

In the conventional adaptive noise cancellation method, the adaptive filter was adjusted to make the output signal y close to the noise n0 to suppress or attenuate the noise n0. In our study, we made some modifications based on the conventional adaptive noise cancellation, as shown in Fig. 3. We built an adaptive filter to filter out the component of the CGO signal, such as the fundamental, and the second and third harmonics. The structure in the red-dotted box was the same as that of the conventional adaptive noise cancellation. The largest difference between the proposed and conventional adaptive noise cancellation methods is the choice of the reference signal. The main channel used the measured Peso signal as its input (p, CGO-contaminated Peso). However, the superposition of the measured Peso signal (p) and constructional signal (ns) was input to the reference channel, as shown by the red-dotted box in Fig. 3. The ns signal was constructed using the sine function whose frequency was based on the heart rate, as mentioned above. From the mechanism of the adaptive filter, the output y is expected to be almost the same as the signal p. Thus, the adaptive filter could eliminate signals that had the same frequency spectrum as the constructional signal (ns). Once the adaptive filter was constructed, the measured Peso signal was input into the same adaptive filter. Thus, the CGO signals can be eliminated from the measured Peso signal as these have the same frequency spectrum as the ns signal. Consequently, the denoised Peso signal (P_filt_) can be obtained. In this study, the filter order was set to 45.

### Normalized LMS algorithm

The LMS algorithm was proposed by Widrow and Hoff in 1959 [27, 28] after studying the pattern recognition scheme of adaptive linear elements. The LMS algorithm has the advantages of low-computational complexity, good convergence in a stationary environment, convergence of mean to the unbiased Wiener solution, stable performance, and simple structure. Therefore, it is the most extensively used adaptive algorithm.

A typical LMS algorithm is described as follows.

1 $$ y\left( n \right)\, = \,W^{T} \left( n \right)X\left( n \right) $$

2 $$ e\left( n \right)\, = \,d\left( n \right)\, - \,y\left( n \right) $$

3 $$ W\left( {n\, + \,1} \right)\, = \,W\left( n \right)\, + \,\mu e\left( n \right)X\left( n \right) $$

where y is the filtered output signal, X is the input signal of the reference channel, W is the definition of a vector of filter coefficients, e is the error signal, d is the input signal of the main channel, μ is the step size for updating the filter coefficients, and the filter order L is assumed to be a sufficiently large constant.

The disadvantage of the classical LMS algorithm is its slow convergence speed. To improve the convergence speed and performance of the LMS algorithm, in this study, we used the normalized LMS algorithm [29], which employs a variable step size method to shorten the adaptive convergence process based on the basic idea of the LMS algorithm. It uses the instantaneous squared error as a simple estimate of the mean-squared error (MSE). Additionally, by controlling the offset, we can obtain the iterative formula for modifying the filter coefficient, considering that the derivative based on the instantaneous squared error is not equal to the derivative of the MSE, as follows.

4 $$ W\left( {n + 1} \right)\, = \,W\left( n \right)\, + \,\frac{\mu }{{\gamma \, + \,X^{T} \left( n \right)X\left( n \right)}}e\left( n \right)X\left( n \right) $$

The variable step size can be represented by μ(n), as follows.

5 $$ \mu \left( n \right)\, = \,\frac{\mu }{{\gamma \, + \,X^{T} \left( n \right)X\left( n \right)}} $$

where γ is a constant (0 ≤ γ ≤ 1), which is set to avoid the step size μ from being too large when the is too small. To ensure that the adaptive filter can stably work, the relationship 0 < μ < 2 must apply.

### Heart-rate-based reference signal

The reference signal can be constructed by using the following equation.

6 $$ n_{s} \left( t \right)\, = \,\sum C_{m} \left( t \right)\, = \,\sum A_{m} *{\text{sin}}\left( {\frac{2\pi *HH*m}{{60}}*t} \right) $$

where m = 1, 2, 3, …., Am is a constant, HH is the heart rate, and t is the time. When m = 1, ns contains only the fundamental frequency. When m = 2, the ns comprises the fundamental and second harmonic frequencies. When m = 3, ns comprises the fundamental, second, and third harmonic frequencies, and so on. In this study, Am was 10, which is the same order of magnitude as the amplitude of the Peso signal.

### Lowpass filter

The lowpass filter was implemented as a linear phase FIR digital filter in Matlab. 0.7 Hz was chosen as the cutoff frequency since most of the low frequency spectral content in the Peso signal stayed below 0.7 Hz in the patient. The filter order was set at 45 which is the same as the proposed method.

### Retrospective clinical data

Retrospective clinical data were collected from 20 (6 female) patients who received ventilator support (SV800, Mindray, Shenzhen, China) in the ICU of the Shenzhen Second People’s Hospital during the period from March 10, 2022, to August 10, 2022. In our study, we included patients with postoperative (abdominal surgery or orthopedic surgery) or acute respiratory failure who were receiving invasive mechanical ventilation. The exclusion criteria were as follows: (1) age < 18 or > 85 years; (2) sedation level on the Richmond Agitation–Sedation Scale ≥ 2; (3) evidence of arrhythmia; (4) contraindication for nasogastric tube insertion, e.g., history of esophageal varices, gastroesophageal surgery in the previous 12 months, or gastroesophageal bleeding in the previous 7 days, international standard ratio > 1.5, activated partial thromboplastin time > 44 s, history of leukemia; (5) hemodynamic instability (heart rate > 140 beats/min, vasopressors required with ≥ 5 μg/kg/min dopa-mine/dobutamine, or ≥ 0.2 μg/kg/min norepinephrine). The data included the Peso, airflow, heart rate, respiration rate. The sample frequency used for collecting and processing the data (including Peso signals and airflow) was 50 Hz. The correct position of the esophageal balloon (SDY-1, Mindray, Shenzhen, China) was verified prior to data collection by a standard occlusion test [12]. We don’t have access to information that could identify individual participants during or after data collection. Patient information is listed in Table 1.

**Table 1:** Patient characteristics (*ARDS* acute respiratory distress syndrome, *COPD* chronic obstructive pulmonary diseases, *SAH* subarachnoid hemorrhage, *PSV* pressure support ventilation, *PEEP* positive end-expiratory pressure, *VCV* volume control ventilation, *PCV* Pressure Controlled Ventilation, *CMV* Controlled mechanical ventilation, *AV* Assisted ventilation)

Patient	Diagnosis	Gender	Ventilator mode	Heart rate (min^−1^)	Respiratory rate (min^−1^)	CMV or AV
1	ARDS	Male	PSV 13 cm H_2_O + PEEP 6 cm H_2_O	84	24	AV
2	ARDS	Male	PCV 14 cm H2O + PEEP 7 cm H2O	118	12	CMV
3	Hemophagocytic syndrome	Female	PSV 12 cm H_2_O + PEEP 6 cm H_2_O	102	21	AV
4	Cerebral infarction	Male	PCV 9 cm H_2_O + PEEP 5 cm H_2_O	119	12	CMV
5	ARDS	Female	PSV 13 cm H_2_O + PEEP 6 cm H_2_O	80	15	AV
6	COPD	Male	PSV 26 cm H_2_O + PEEP 12 cm H_2_O	78	10	AV
7	After surgery	Male	PSV 14 cm H_2_O + PEEP 6 cm H_2_O	84	19	AV
8	After surgery	Male	PSV 14 cm H_2_O + PEEP 7 cm H_2_O	81	21	AV
9	COPD	Female	PSV 13 cm H_2_O + PEEP 6 cm H_2_O	102	18	AV
10	After surgery	Male	PSV 20 cm H_2_O + PEEP 9 cm H_2_O	90	13	AV
11	ARDS	Male	PSV 22 cm H_2_O + PEEP 6 cm H_2_O	106	24	AV
12	After surgery	Male	PSV 18 cm H_2_O + PEEP 6 cm H_2_O	68	17	AV
13	COPD	Male	PSV 25 cm H_2_O + PEEP 6 cm H_2_O	81	17	AV
14	After surgery	Male	PSV 12 cm H_2_O + PEEP 6 cm H_2_O	85	16	AV
15	Sepsis	Male	VCV 440 mL	79	12	CMV
16	Severe craniocerebral injury	Male	VCV 450 mL	72	14	CMV
17	Traumatic brain injury	Male	VCV 460 mL	80	12	CMV
18	SAH	Female	VCV 400 mL	111	12	CMV
19	Sepsis	Female	PCV 16 cm H_2_O + PEEP 6 cm H_2_O	97	14	CMV
20	Severe pneumonia	Female	PCV 10 cm H_2_O + PEEP 16 cm H_2_O	100	10	CMV

### Method used to compute noise intensity

The noise intensity can be quantified by the average peak-to-peak value of five segments of flat transpulmonary pressure. The transpulmonary pressure was calculated as the difference between the airway pressure and the esophageal pressure.

### Method of filter performance verification

**Fig. 4:**
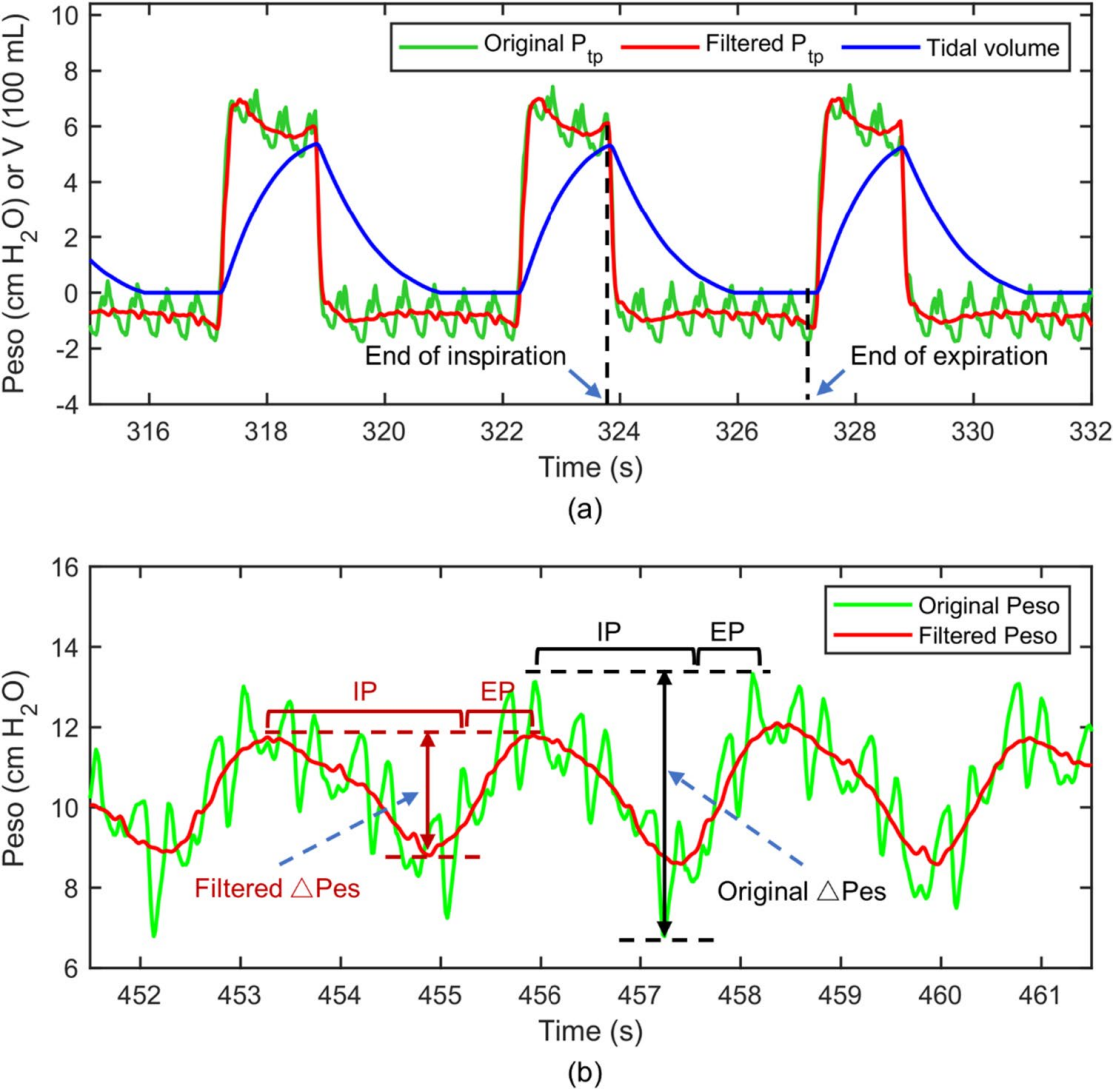
**a** Schematic of end-inspiration and end-expiration point selection. **b** Schematic of original △Pes and filtered △Pes calculation. The beginning of the inspiratory phase was identified at the time of Pes initial decay, whereas the end of inspiration was considered at the point of Pes that elapsed 25% of time from its maximum deflection to return to baseline. *IP* Inspiratory phase, *EP* Expiratory phase, *P*_*tp*_ Transpulmonary pressure, *V* Tidal volume

The main goal of this section was to evaluate the stability and dependability of Peso. QUOTE can be used to confirm the stability of the Peso measurement and compare directly the amplitude shifts and frequency spectra of the original Peso with the filtered Peso signal. For each respiratory cycle, QUOTE has a single value, and in patients with controlled mechanical ventilation, it is expressed as,

7 $$ C_{lung} \, = \,{ }\frac{V}{{P_{tp\_I} \, - \,P_{tp\_E} }} $$

where V is the tidal volume, is the transpulmonary pressure (Ptp) value at the end of inspiration, and is the Ptp value at the end of expiration, as shown in Fig. 4a. The Ptp value is calculated using the airway pressure subtracted from the Peso.

The stability and dependability of Peso were confirmed by using the standard deviation and fluctuation of . The mean values of from the original and processed data were compared to prevent signal distortion during the filtering process. In theory, the compliance of the lung should be relatively constant. The standard deviation of will be low if the value of does not change considerably. However, the value of will fluctuate considerably if the heartbeat artifact in Peso is large. Therefore, the stability of can be utilized to assess the effectiveness of the filter.

The △Pes plays an important role in assessing respiratory muscle strength, monitoring intrathoracic pressure, and evaluating ventilator settings, which helps determine the optimal timing for ventilator support and weaning, and can also prevent the occurrence of complications. It is calculated by using the Peso value at the beginning of the inspiratory phase subtracted from the peak value of the Peso signal in the same respiratory cycle in patients with assisted ventilation [30], as shown in Fig. 4b.

The technique was implemented in MATLAB (The MathWorks Inc., Natick, MA, USA) with a computer operating on a 64-bit Windows 10 Professional operating system, 16 GB RAM, and a 3.00 GHz Intel(R) Core (TM) i5-9500 CPU.

### Statistical analysis method

Statistical analysis of multiple comparisons was performed using the Mann–Whitney U test. The student's t-test statistic was applied for the analysis of standard deviation (std)/mean of QUOTE and △Pes, and reduction of std/mean of △Pes, in the original and filtered Peso groups. The comparison between the proposed method and lowpass filter was also analyzed using the Student's t-test statistic. P values < 0.05 denote statistical significance.

## Results

**Fig. 5:**
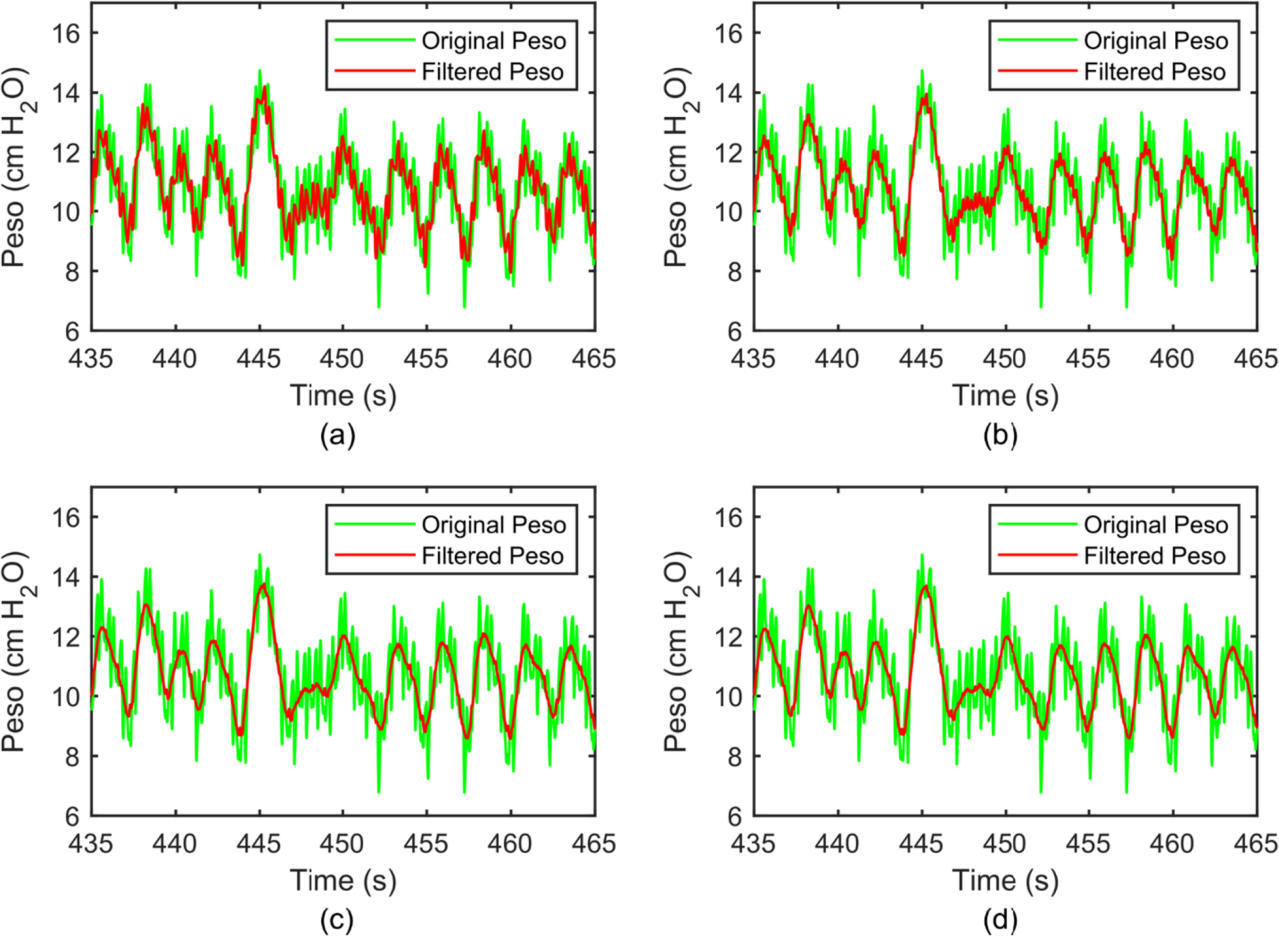
Original Peso (green color) and the filtered Peso signals (red color) with m = 1 (**a**), m = 2 (**b**), m = 3 (**c**), and m = 4 (**d**) in the time domain

**Fig. 6:**
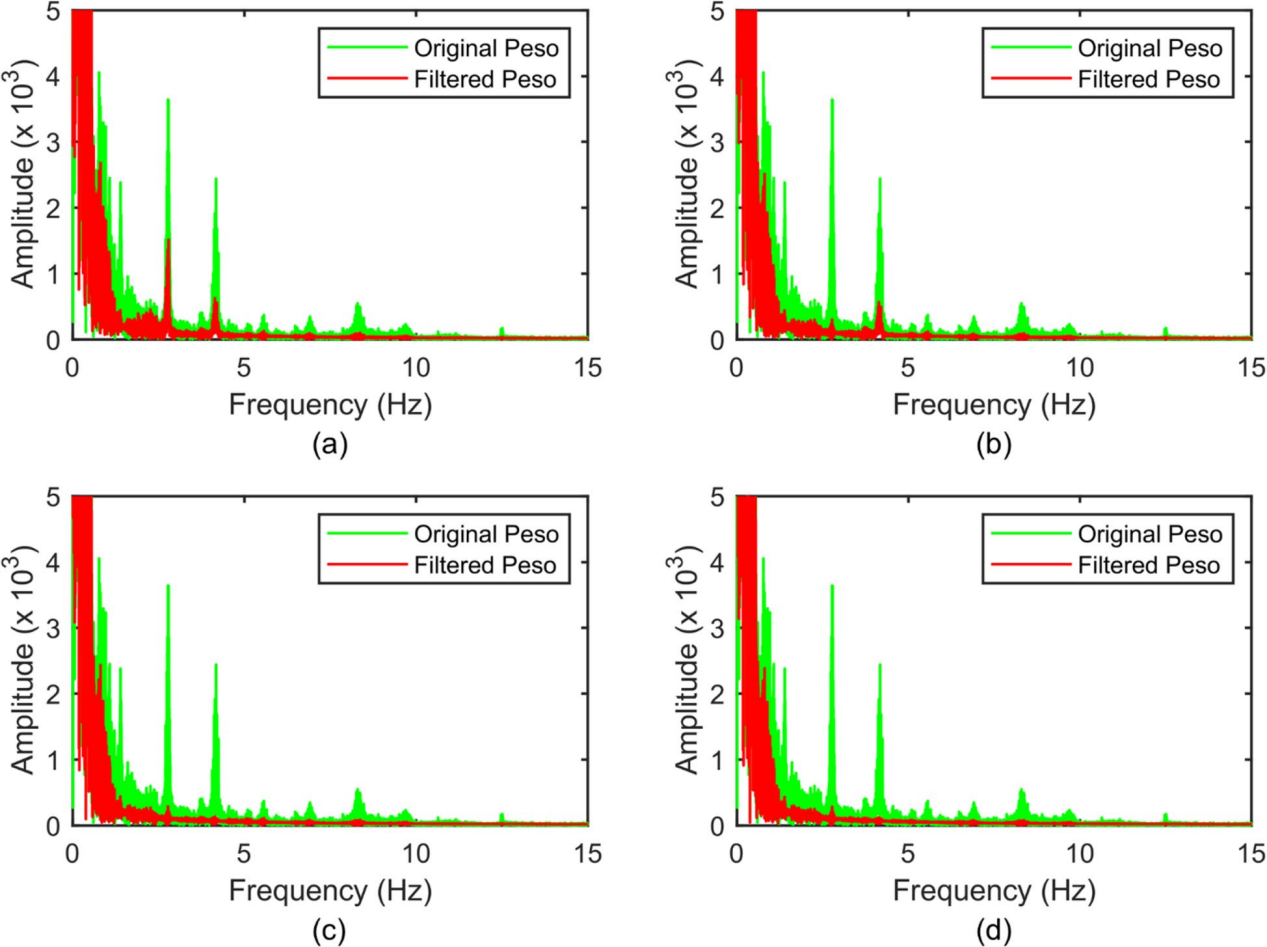
Frequency spectra of the original Peso (green color) and the filtered Peso signals (red color) with m = 1 (**a**), m = 2 (**b**), m = 3 (**c**), and m = 4 (**d**)

Figure 5 shows one sample of the original Peso signal (green color) and the filtered signals (red color) with different reference signals. We can see that the waveform of the original Peso was mixed with other higher-frequency signals. From its frequency spectrum, shown by the green color in Fig. 6, the frequency of the peak is at 1.4 Hz, and corresponding to a heart rate at 84 beats per minute. Therefore, the frequencies of the peak at 2.4 Hz and 4.8 Hz correspond to the second and third harmonic frequencies of the CGO signal, respectively.

**Fig. 7:**
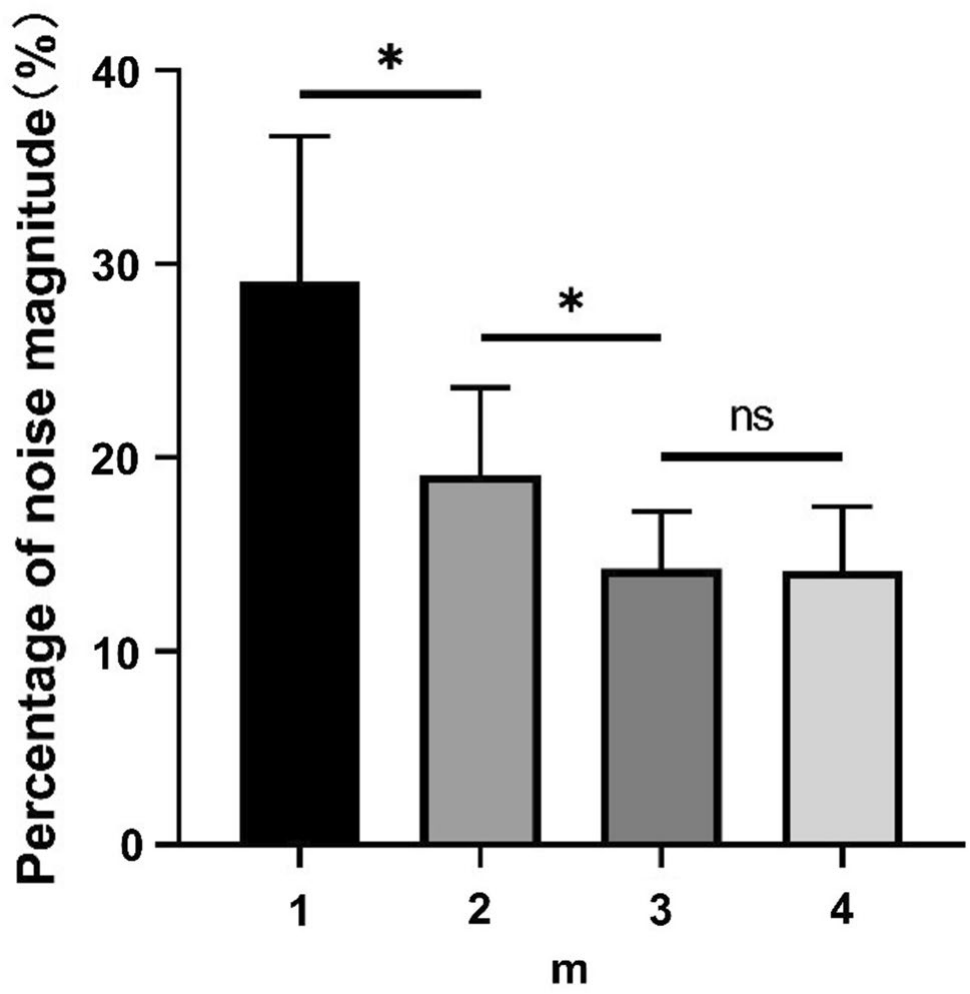
Percentages of filtered noise with the use of different reference signals (m = 1, 2, 3, 4) compared with the noise of the original signal (n = 5). The P values of m = 1 vs. m = 2, m = 2 vs. m = 3, and m = 3 vs. m = 4 are 0.026, 0.014, 0.83 respectively. Herein, the symbol * denotes statistically significant difference, “ns” denotes no significant difference

When m = 1, some of the CGO-related noise was suppressed, but noise still existed (amplitude approximately equal to 1 cmH2O) in the filtered Peso signal, as shown in the red color in Fig. 5a. The frequency domain of the original and filtered Peso signals (m = 1), shown in Fig. 6a, reveal that the fundamental frequency of the CGO was eliminated, but a part of the second and third harmonic frequencies of the CGO signal remained. When m = 2, some CGO-related noise remained (with intensity approximately equal to 0.6 cmH2O) as shown in Fig. 5b. In its frequency domain, as shown in Fig. 6b, both the fundamental and second harmonic frequencies of the CGO were filtered, but the third harmonic frequency still existed. When m = 3, the CGO-related noise was negligibly eliminated, as shown in Fig. 5c, with the remaining noise intensity in the range of 0.2–0.3 cmH2O. This was also demonstrated in the frequency domain, as shown in Fig. 6c. Moreover, when m = 4, the same denoising results as those for m = 3 were obtained, as shown in Figs. 4d and 5d. In Fig. 7, when m = 1, 2, 3, and 4, 27.7 ± 9.7%, 19.1 ± 4.4%, 14.3 ± 2.9%, and 14.2 ± 3.2% of the original CGO-related noise remained, respectively. The P values of m = 1 vs. m = 2, m = 2 vs. m = 3, and m = 3 vs. m = 4 are 0.026, 0.014, 0.83 respectively. Evidently, there are not considerable differences between the m = 3 and m = 4 cases observed. This indicates that setting m = 3 is sufficient to remove the CGO-related noise. Therefore, in the following study, the value of m was set to three to construct the reference signal.

**Fig. 8:**
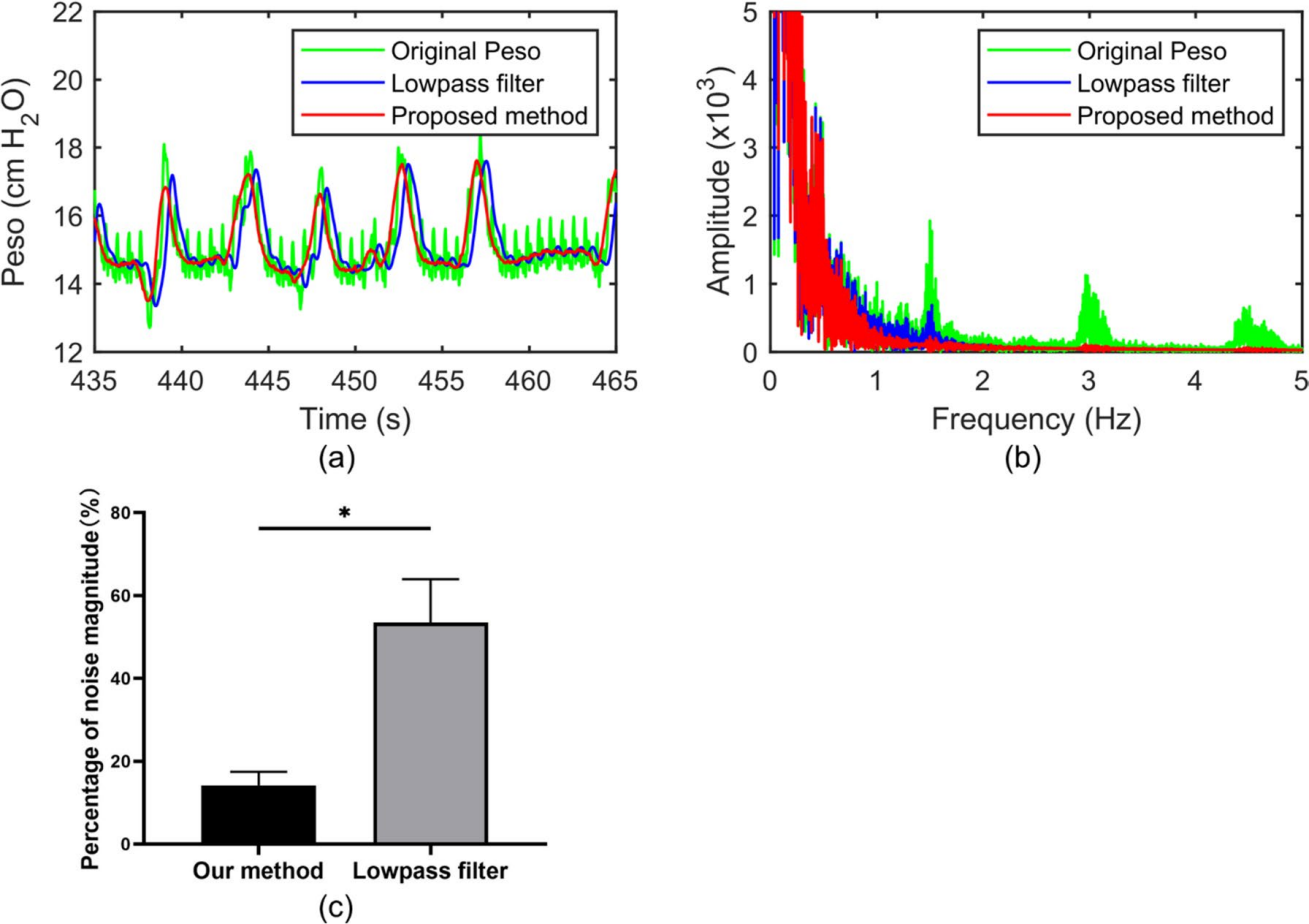
Three types of Peso signal (green color: original; blue color: filtered by lowpass filter; red color: filtered by the proposed method) in both the time (**a**) and frequency domains (**b**). **c** The Percentages of noise magnitude of the proposed method group and the lowpass filter group. The symbol * denotes statistically significant difference

When we simply applied a lowpass filter with a 1 Hz cutoff frequency and 45 filter order to process the Peso signal, it remained with 0.44 ± 0.07 cm H20 fluctuation caused by the CGO, as shown in the blue line in Fig. 8a. This can be further confirmed by the frequency analysis, as it retained a part of the fundamental frequency of the CGO signal, as shown in the blue line in Fig. 8b. However, with the same filter order, our proposed method can remove the CGO more effectively, leaving only 0.19 ± 0.06 cm H20 of CGO-related noise, as shown in the red line in Fig. 8a. Its frequency spectrum also shows that the CGO component was cleanly suppressed, as shown in the red line in Fig. 8b. According to the quantitative analysis, only 11.42% ± 2.75% of noise is left behind after the Pes signal is processed by the proposed method. In the Lowpass filter group, it retained 26.68% ± 3.03% of CGO-related noise, as shown in Fig. 8b.

**Fig. 9:**
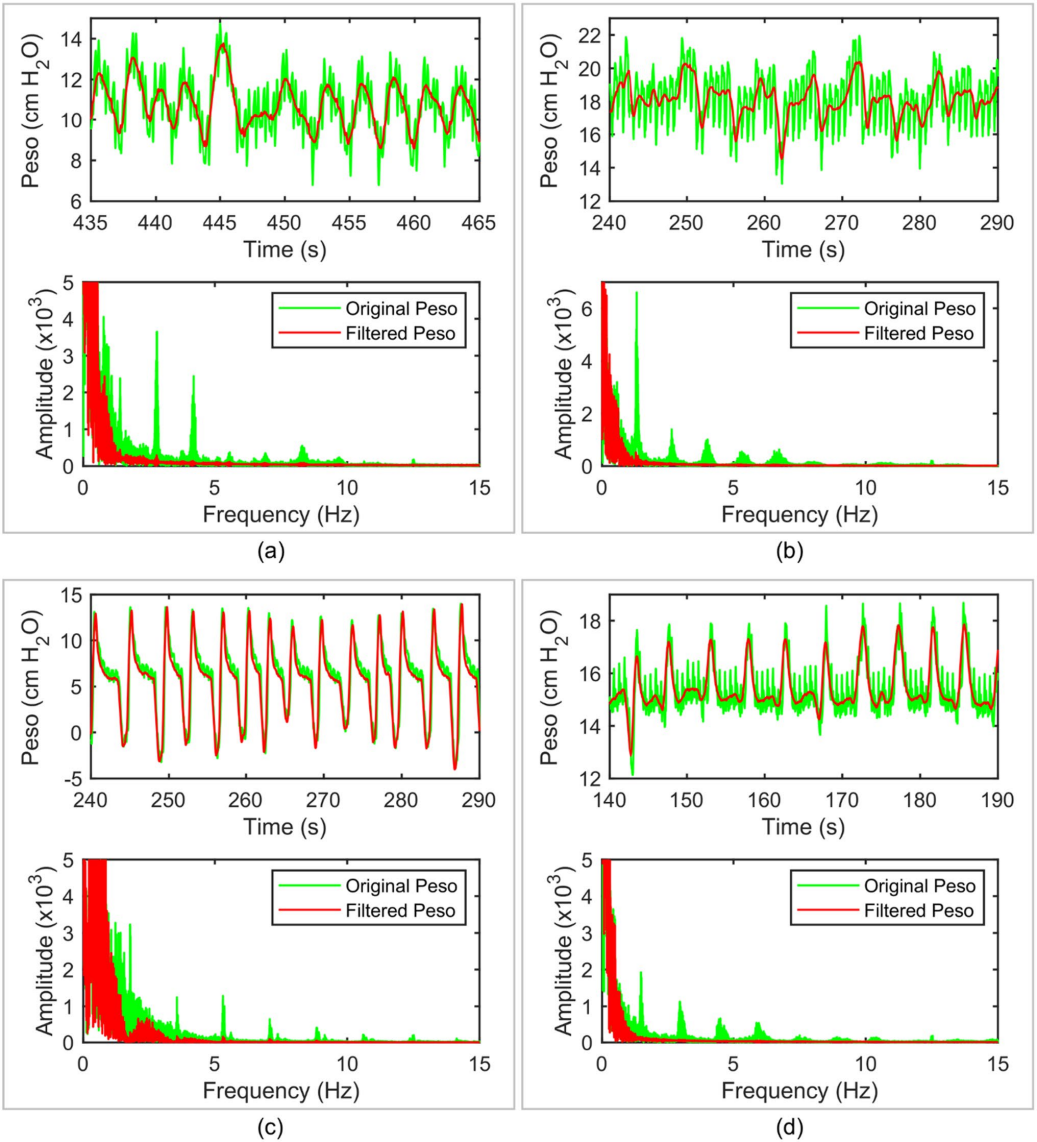
Original and filtered (m = 3) Peso signals in both the time and frequency domains obtained from the four studied patients. **a** Patient #1 (acute respiratory distress syndrome (ARDS)), **b** Patient #5 (ARDS), **c** Patient #9 (chronic obstructive pulmonary diseases (COPD)), and **d** Patient #10 (postsurgical state)

Figure 9 shows samples of the original (green color) and filtered (red color) Peso signals and the corresponding frequency spectra of four ICU patients with different diagnoses and heart rates. In all four graphs, the coefficient of the reference signal was m = 3. Patients A and B were diagnosed with acute respiratory distress syndrome, and their heart rates were 84 and 80 beats per minute, respectively. Patient C was diagnosed with chronic obstructive pulmonary disease with a heart rate of 102 beats per minute. Patient D had a heart rate of 90 beats per minute and was in a postsurgical state. All the filtered Pes signals of the four studied patients demonstrated effective removal of CGO-related noise. Additionally, although the remaining frequencies remained intact, the filter reduced the peaks of Peso in the Fourier spectrum at integer multiples of the heart rate. This demonstrates that our technique can effectively denoise the CGO-related noise in different situations, such as at different patient heart rates and diseases.

**Fig. 10:**
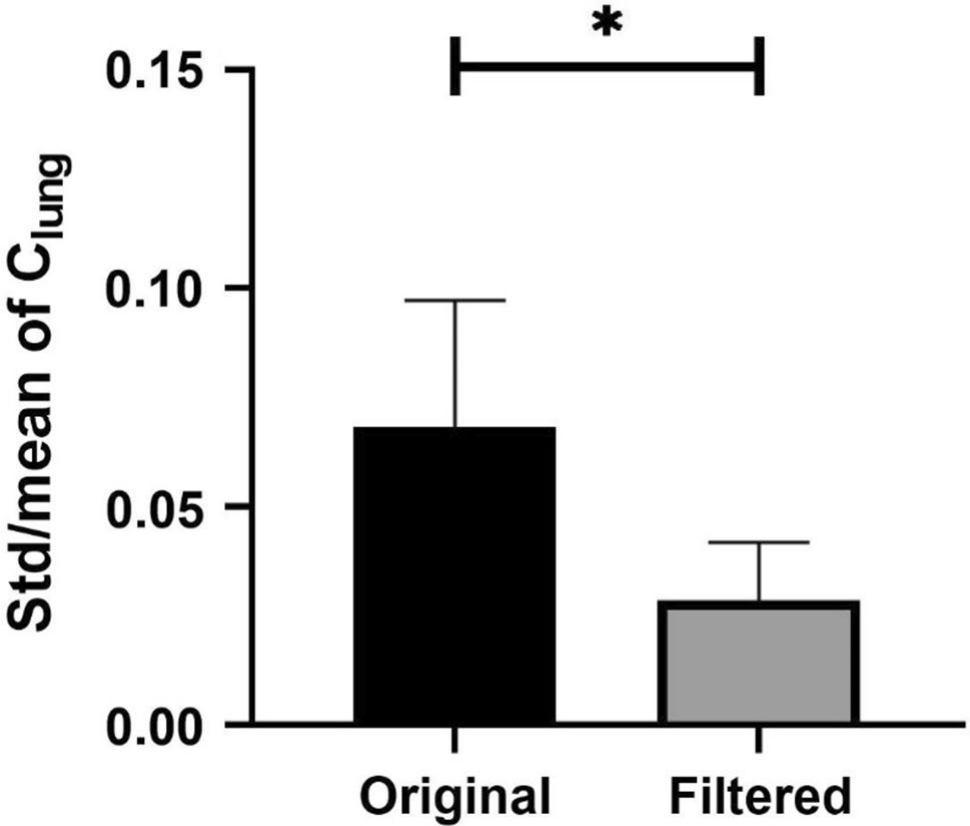
Statistical analysis of standard deviation (std)/mean of in the original and filtered Peso groups (n = 8). The symbol * denotes statistically significant difference

Table 2 lists the mean and standard deviation (std) of the for all the patients (patients 2, 4, 15–20) with controlled mechanical ventilation. In all the 8 patients, the mean of the original and filtered data are considered the same (P > 0.05) and the std/mean of the filtered data is much less than that of the original data. The standard deviation of the std/mean of was reduced by 28.4–79.2%. The mean of std/mean of reduced from 0.069 to 0.029 (60.0% reduction) after filtering, as shown in Fig. 10. This suggests that the proposed technique can suppress the CGO signal without impairing the Peso signal.

**Table 2:** values obtained from the original and filtered Peso signal (mean ± standard deviation (std)) in controlled mechanical ventilation

Patient	Number of C_lung_	C_lung_ (original Peso)		C_lung_ (filtered Peso)		Reduction of std/mean (%)	P value (t-test)
		Mean ± std, mL/cm H_2_O	Std/mean	Mean ± std, mL/cm H_2_O	Std/mean		
2	30	36.53 ± 1.82	0.050	36.71 ± 0.47	0.015	69.1	0.606
4	30	78.22 ± 6.94	0.089	77.27 ± 1.42	0.018	79.2	0.466
15	30	31.6 ± 2.35	0.074	30.92 ± 1.08	0.035	53.1	0.153
16	30	54.76 ± 5.37	0.098	54.18 ± 2.23	0.041	58.1	0.587
17	30	29.66 ± 0.53	0.018	29.47 ± 0.38	0.013	28.4	0.131
18	30	27.16 ± 2.59	0.095	27.06 ± 1.27	0.047	50.8	0.859
19	30	36.80 ± 1.58	0.043	36.18 ± 0.81	0.022	47.9	0.064
20	30	11.91 ± 0.96	0.081	11.90 ± 0.47	0.039	51.4	0.951

**Fig. 11:**
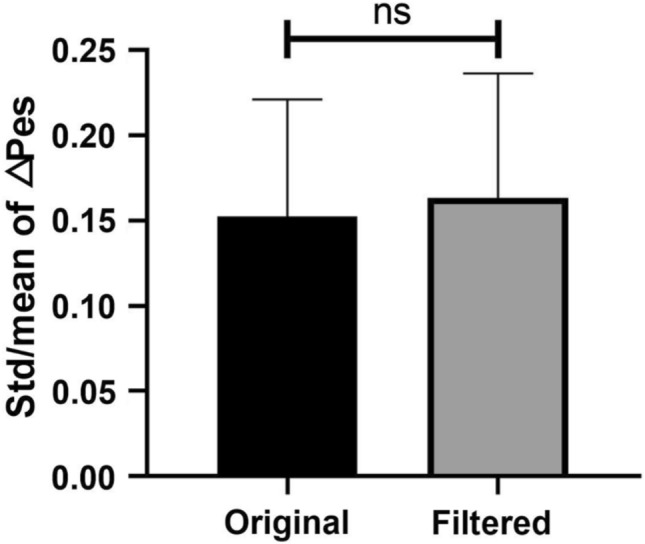
Statistical analysis of std/mean of △Pes in original and filtered Peso groups (n = 12). Herein, “ns” denotes no significant difference

The △Pes of the original Peso was higher than the △Pes of the filtered Peso in all 12 patients (patients 1, 3, 5–14) with assisted ventilation, as shown in Table 3. A reduction in △Pes of approximately 38.7 ± 11.4% after filtering was observed, which suggests that 38.7 ± 11.4% of the original △Pes was the error caused by the CGO signal. The filtered signal can provide us with a more accurate △Pes for the assessment of spontaneous effort. However, no significant difference in the std/mean of△Pes between the original and filtered signals was observed, as shown in Fig. 11. This suggests that the fluctuation of the CGO signal was constant during the Peso measurement. This fluctuation will amplify the absolute value of △Pes, thereby causing an error in the assessment of the spontaneous effort.

**Table 3:** Esophageal pressure swings (△Pes) of the original and filtered Peso signals (mean ± std) in assisted ventilation

Patient	Number of Pes	△Pes (original Peso)		△Pes (filtered Peso)		Reduction of mean (%)	Reduction of std/mean (%)	P value (t-test)
		Mean ± standard deviation (std)	Std/mean	Mean ± std	Std/mean			
1	30	− 8.84 ± 2.55	0.289	− 5.89 ± 1.87	0.317	33.36	− 9.81	< 0.001
3	30	− 2.05 ± 0.21	0.102	− 1.09 ± 0.13	0.119	46.83	− 16.67	< 0.001
5	30	− 6.72 ± 1.50	0.223	− 4.25 ± 0.85	0.200	36.69	10.00	< 0.001
6	30	− 6.16 ± 0.73	0.119	− 4.54 ± 0.53	0.116	26.34	2.35	< 0.001
7	30	− 5.20 ± 0.40	0.076	− 2.68 ± 0.17	0.062	48.45	18.41	< 0.001
8	30	− 9.79 ± 0.95	0.097	− 4.34 ± 0.68	0.157	55.61	− 62.52	< 0.001
9	30	− 9.04 ± 0.96	0.106	− 6.23 ± 0.69	0.111	31.15	− 4.05	< 0.001
10	30	− 4.23 ± 0.42	0.099	− 2.72 ± 0.27	0.101	35.64	− 1.50	< 0.001
11	30	− 5.55 ± 0.66	0.119	− 2.36 ± 0.31	0.132	57.57	− 10.60	< 0.001
12	30	− 5.44 ± 1.19	0.218	− 4.39 ± 0.97	0.222	19.45	− 1.72	< 0.001
13	30	− 3.21 ± 0.51	0.157	− 2.06 ± 0.35	0.169	35.72	− 7.09	< 0.001
14	30	− 2.21 ± 0.50	0.225	− 1.38 ± 0.35	0.254	37.51	− 12.85	< 0.001

**Fig. 12:**
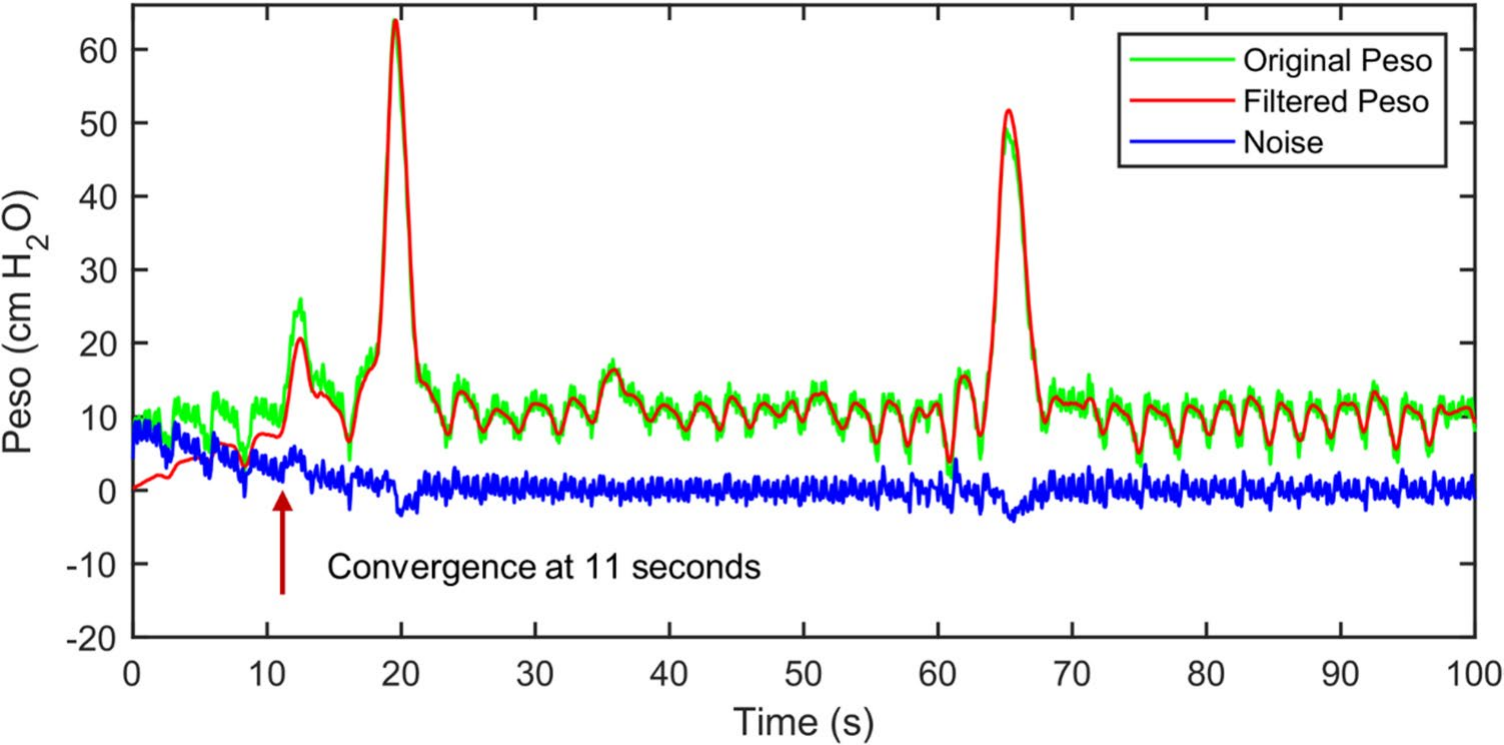
Real-time filtering of a Peso signal

Figure 12 shows the original and filtered Pes signals, as well as the noise calculated by subtracting the filtered Pes signal from the original Pes signal during the filtering process. Evidently, the filtering process converged at 11 s.

## Discussion

The current study provided a detailed description of a modified adaptive filter that effectively suppressed the CGOs that complicated Peso signal processing. Primarily, this technique uses a heart rate-based signal for the reference signal instead of the ECG, or EMG, or another type of signal, which require additional equipment. The CGO signal was effectively suppressed without impairing the Peso signal when the reference signal contained the fundamental, second, and third harmonic frequencies of the CGO. The presence of CGO noise leads to an overestimation of the original measurement of △Pes. An overestimated △Pes can have misleading effects on predicting weaning from non-invasive ventilation, potentially leading to premature extubation, an increased risk of patients requiring reintubation, worsening patient condition, and an elevated risk of mortality. By utilizing the proposed technique, a more accurate measurement of △Pes can be achieved. The proposed technique can also be easily adapted for denoising other biomedical signals, such as the ECG and photoplethysmogram, which have either a periodic or quasiperiodic nature. In the frequency domain, peaks in the Fourier spectrum of Peso at integer multiples of the heart rate were suppressed without affecting the remaining frequencies. This result corroborates Cheng’s research, which indicated that the fourth or higher harmonics had minimal impact on the filter's performance [17]. Since the reference signal is based on the heart rate, there is a concern that when the respiratory rate and its multiples (e.g., 2 × and 3 ×) align with the frequency of the fundamental (and the second and third harmonics) of the CGO signal, the filtered Peso signal may be somewhat distorted. Although the adaptive filter convergence required 11 s, the heart rates of most patients were stable most of the time during their monitoring in the clinic, and the filtered Peso signals were accurate and reliable most of the time. Only when the patient's heartbeat significantly fluctuated over a specific period, the adaptive filter required additional time for convergence during real-time monitoring. To make this technique suitable for more complex situations, we can work on optimizing the adaptive filtering algorithm to shorten the convergence time of the adaptive filter in the future. Some researchers have previously developed methods that effectively filter out CGO signals. However, some of these methods require the use of additional reference signals for filtering, such as Schuessler et al. [18], Cheng et al. [1, 17], and Graßhoff et al. [4]. To achieve good filtering results, it is necessary to ensure that the reference signal and the esophageal pressure signal are synchronized. The introduction of additional reference signals also increases the cost for patients and the workload of healthcare workers. Some methods also require a large amount of computing resources and time, such as Mukhopadhyay et al. [19], making it impossible to perform real-time filtering of the esophageal pressure signal and provide timely feedback information to healthcare workers to help adjust the patient's ventilation settings. These issues largely limit the clinical application of these methods. However, our method does not require additional reference signals and can filter out CGO noise in real-time while providing accurate esophageal pressure signals. This will provide useful esophageal pressure information to healthcare professionals for timely adjustment of the patient's ventilation parameters. It is clear that this technology has great value in clinical applications. With the advantages of not requiring additional signals as reference signals and compressing the CGO noise in real-time, the proposed technology can be easily and effectively used in clinical settings before long.

## Conclusions

In summary, we have successfully developed a modified adaptive filter to suppress the CGO signals, where the reference signal was constructed based on the heart rate using a sine function. When the reference signal included the fundamental, second, and third harmonic frequencies of the CGO, the CGO signal was effectively eliminated from the original Peso signal. However, when the reference signal contained the fourth or higher harmonic frequencies of the CGO, there was no significant improvement in the filter's performance. We validated the clinical performance of our technique by assessing lung compliance and △Pes. Our proposed technique significantly reduced the std/mean of QUOTE and improved the accuracy of △Pes measurements. These findings demonstrate that our technique is highly efficient and enhances the precision of lung compliance and △Pes measurements.
